# Oxytocin receptor antagonists as a novel pharmacological agent for reducing smooth muscle tone in the human prostate

**DOI:** 10.1038/s41598-021-85439-4

**Published:** 2021-03-18

**Authors:** Sophie N. Lee, Jenna Kraska, Melissa Papargiris, Linda Teng, Birunthi Niranjan, Johanna Hammar, Andrew Ryan, Mark Frydenberg, Nathan Lawrentschuk, Ralf Middendorff, Stuart J. Ellem, Michael Whittaker, Gail P. Risbridger, Betty Exintaris

**Affiliations:** 1 Department of Anatomy and Developmental Biology, Biomedicine Discovery Institute, Monash University, Clayton, VIC Australia; 2 Drug Discovery Biology, Monash Institute of Pharmaceutical Sciences, Monash University, 381 Royal Parade, Parkville, Melbourne, VIC 3052 Australia; 3 TissuPath, Melbourne, VIC Australia; 4 Department of Surgery, Monash University, Melbourne, VIC Australia; 5 Australian Urology Associates, Melbourne, VIC Australia; 6 Department of Surgery, Austin Health, University of Melbourne, Melbourne, VIC Australia; 7 EJ Whitten Prostate Cancer Research Centre at Epworth Heathcare, Melbourne, Australia; 8 Institute of Anatomy and Cell Biology, Justus-Liebig-University Giessen, Giessen, Germany; 9 School of Health and Wellbeing, Faculty of Health, Engineering and Sciences, University of Southern Queensland, Ipswich, QLD Australia; 10 ARC Centre of Excellence in Convergent Bio-Nano Science and Technology, Faculty of Pharmacy and Pharmaceutical Sciences, Parkville, VIC Australia

**Keywords:** Endocrinology, Urology

## Abstract

Pharmacotherapies for the treatment of Benign Prostatic Hyperplasia (BPH) are targeted at reducing cellular proliferation (static component) or reducing smooth muscle tone (dynamic component), but response is unpredictable and many patients fail to respond. An impediment to identifying novel pharmacotherapies is the incomplete understanding of paracrine signalling. Oxytocin has been highlighted as a potential paracrine mediator of BPH. To better understand oxytocin signalling, we investigated the effects of exogenous oxytocin on both stromal cell proliferation, and inherent spontaneous prostate contractions using primary models derived from human prostate tissue. We show that the Oxytocin Receptor (OXTR) is widely expressed in the human prostate, and co-localises to contractile cells within the prostate stroma. Exogenous oxytocin did not modulate prostatic fibroblast proliferation, but did significantly (*p* < 0.05) upregulate the frequency of spontaneous contractions in prostate tissue, indicating a role in generating smooth muscle tone. Application of atosiban, an OXTR antagonist, significantly (*p* < 0.05) reduced spontaneous contractions. Individual tissue responsiveness to both exogenous oxytocin (R^2^ = 0.697, *p* < 0.01) and atosiban (R^2^ = 0.472, *p* < 0.05) was greater in tissue collected from older men. Overall, our data suggest that oxytocin is a key regulator of inherent spontaneous prostate contractions, and targeting of the OXTR and associated downstream signalling is an attractive prospect in the development of novel BPH pharmacotherapies.

## Introduction

Benign Prostatic Hyperplasia (BPH) is an extremely common disorder of the ageing male population, resulting from dysregulated proliferation and hypertrophy of the prostate stroma^1^. The resultant increase in pressure surrounding the urethra contributes to the formation of Lower Urinary Tract Symptoms (LUTS), which have a significant burden both in cost and quality of life (QoL) in elderly men^2^.

Current pharmacological treatments for BPH aim to either block epithelial/stromal proliferation and inhibit cell survival (5α-reductase inhibitors), or prohibit prostatic contractions and reduce prostatic smooth muscle tone (α1-adrenoreceptor antagonists (α-blockers); Phosphodiesterase Type 5 (PDE5) inhibitors)^3^. Both 5α-reductase inhibitors and α-blockers are known to be clinically effective at reducing LUTS symptoms, however they both have significant impacts independently on ejaculation^4^. In combination, men are threefold more likely to develop an ejaculatory issue following pharmacological treatment^4^. While PDE5 inhibitors have extremely positive effects on ejaculation^5^, responsiveness to PDE5 inhibitors is unpredictable, and highly dependent on patient parameters, including age, body mass index, and symptom severity prior to commencing therapy^6^. As erectile dysfunction is a key component of QoL, and is known to significantly contribute to anxiety and depression in older men^7^, there is an onus on developing new and novel therapies for the treatment of BPH that do not negatively impact erectile function.

A possible novel paracrine target for pharmaceutical treatment of BPH is the hormone oxytocin. Oxytocin is well described in the female reproductive system for its role in the uterus and pregnancy, where oxytocin induces myometrial contractions through its G-protein coupled receptor, the oxytocin receptor (OXTR). Activation of the OXTR in the uterus through the Gα_q/11_-subunit leads to the downstream activation the IP3/DAG signalling pathway, leading to smooth muscle contraction^8^. Previous studies have indicated that glandular regions of the prostate are immunoreactive for oxytocin, with staining for OXTR observed in both glandular and stromal regions of the prostate^9,10^. Herbert et al. reported that the expression of the OXTR increased with age and was higher in the BPH tissue^11^. There is some evidence that oxytocin induces contraction within the human prostate. A number of studies report oxytocin inducing contraction of human, guinea pig and dog prostate tissue^12,13^. However, a study conducted by Gupta et al. failed to induce contraction of the rat prostate in response to oxytocin^14^, raising the question of differing responses between species of mammal.

Other G-protein subunits of the OXTR are linked to both proliferative and anti-proliferative pathways in different organs^15^. The role of oxytocin on prostate proliferation has yet to be fully elucidated, with conflicting reports that oxytocin is able to regulate proliferation of stromal and epithelial cells^10,16^. Whittington et al. demonstrated that incubation of isolated primary prostate fibroblasts with oxytocin significantly reduced proliferation^16^, while, in contrast, Xu et al. showed that incubation of immortalised prostate fibroblasts (WPMY-1) increased proliferation via activation of the MAP/ERK pathway^10^.

To clarify the role of oxytocin within the prostate, we utilised a cohort of human primary prostate specimens to investigate the effects of exogenous oxytocin on both the proliferation and contractility of the prostate, and examine the suitability of atosiban, a commonly clinically utilised OXTR antagonist, as a potential BPH pharmacotherapy.

## Materials and methods

### Patient cohort

Human prostatic tissue was collected with informed written consent from patients and approval from the Cabrini Human Research Ethics Committee (13-14-04-08), Epworth HealthCare Human Ethics Committee (53611) and Monash University Human Research Ethics Committee (2004/145). All experiments were performed in accordance with relevant guidelines and regulations. Tissue was collected from men undergoing radical prostatectomy for the treatment of low to medium grade prostatic cancer (Gleason Grade Group ≤ 3), with patient data listed in Table 1.

**Table 1:** Patient demographics.

Demographic	Number (% of cohort)	Mean (± SD)	Range
Total patients	15 (100)		
Clinical BPH diagnosis	0 (0)		
Minimal LUTS reported	4 (26.67)		
Significant LUTS reported	0 (0)		
Age		67.7 (6.97)	53–75
Prostate volume (cc)		39.46 (9.09)	27–55
Gleason score		7 (N/A)	6–9
Percent tumour volume (tumour volume/total volume)		6.99 (4.06)	1.53–15

### Tissue procurement

Patients were selected prior to surgery based on biopsy results, indicating a low-grade, small volume tumour localised to the Peripheral Zone (PZ) of the prostate. Tissue was collected exclusively from the Transition Zone (TZ) of the prostate, as this is the area most proximal to the urethra, and the zone from which BPH arises. Dissection of non-malignant tissue from the prostate was performed by a board-certified pathologist, with Haematoxylin and Eosin (H&E) staining performed on a small piece of adjacent tissue to ensure no tumour infiltration within the non-malignant section. Tissue for histology was placed in formalin (10%), with tissue for organ bath and cell cultures placed in Physiological Saline Solution (PSS; 2.5 mM CaCl_2_, 11 mM d-Glucose, 5 mM KCl, 120 mM NaCl, 25 mM NaHCO_3_, 1.2 mM KH_2_PO_4_, 1.2 mM MgSO_4_, bubbled with a 95% O_2_:5% CO_2_ gas mixture to maintain a pH of 7.3–7.4), and transfer media [RPMI 1640 with penicillin–streptomycin (P–S), 5% Fetal Calf Serum (FCS), and gentamicin (100 µg/mL)] respectively.

### Immunohistochemistry and tissue stains

Sections were de-paraffinised and rehydrated through a series of xylene and graded ethanol solutions. Sections were placed in 1.2% H_2_O_2_ for 15 min to block endogenous peroxidase activity. For immunohistochemistry, tissue was incubated with the primary antibody OXTR (1 μg/mL; Abcam Cat# ab87312, RRID:AB_10674457), diluted in PBS with 0.2% BSA and 0.1% sodium azide for 24 h at 4 °C. Sections were washed with PBS, and incubated with an anti-goat secondary antibody (1.25 μg/mL), the Vectastain ABC-HRP Kit and DAB + chromogen (DAKO), followed by counterstaining with haematoxylin. For immunofluorescence, sections were incubated with both α-Smooth Muscle Actin (α-SMA) (5.3 μg/mL, Sigma-Aldrich Cat# A2547, RRID:AB_476701) and OXTR, and visualised using a donkey anti mouse IgG Alexa Fluor 488 secondary antibody (1:500, Molecular Probes Cat# A-21202, RRID:AB_141607), and a rabbit anti goat IgG Alexa Fluor 555 secondary antibody (1:500, Thermo Fisher Scientific Cat# A-21428, RRID:AB_2535849). Sections were incubated with DAPI (1:1000, Thermo Fisher Scientific Cat# D3571, RRID:AB_2307445). Negative control staining was performed by incubating sections with Goat IgG (Zymed Laboratories 02-6202) and/or Mouse IgG2a (Dako X094301-2).

### Organ bath studies

Tension recordings were obtained from transition zone (TZ) specimens as previously described^17^.

An initial tension of 25 mN was applied to the tissue, which was then left to equilibrate for 60 min. The ‘basal tension’ describes the tension of the tissue following this equilibration period. This tension was not re-adjusted to a predefined baseline to allow spontaneous contractions to emerge.

#### Reagent preparation

Powdered oxytocin (Sigma Aldrich, St Louis, MO, USA) and atosiban (Sigma Aldrich, St Louis, MO, USA) were dissolved in distilled water to form a 1 mM stock concentration. Serial dilutions were performed by diluting the 1 mM stock into PSS solution.

#### Concentration–response curve to oxytocin

Following the equilibration period, a cumulative concentration–response curve (CDC) to oxytocin (0.1 nM to 10 μM) were constructed by perfusing sections for four (4) minutes with increasing concentrations of oxytocin dissolved in PSS. Following a washout period, the tissue was challenged with a potassium chloride solution (20 mM) to assess tissue viability and reliably induce a robust contraction in all viable preparations. Preparations were subsequently weighed following the completion of experiments.

#### Spontaneous contractions

Following the equilibration period, the preparation was incubated with increasing concentrations of atosiban (1, 10, 100, and 300 nM) prepared in fresh Physiological Saline Solution (PSS), for a period of 10 min at each concentration. Following a washout period, the tissue was challenged with a potassium chloride solution (20 mM) to assess tissue viability. Preparations were weighed following the completion of experiments, with the amplitude of spontaneous contractions (Δ change in tension from baseline) normalised to tissue weight^18^.

#### Analysis of contractile recordings

Analysis of tension recordings focused on three parameters: basal tension (the inherent tension within the tissue; mN), as well as amplitude and frequency (strength and speed of contractions; N/g and min^−1^, respectively) as previously reported^19,20^. These parameters are as used in previous studies to define spontaneous contractions^19,21,22^. Chart Pro v 5.5.6 was used to analyse the tension recordings, with indicated parameters measured for 5 consecutive responses and averaged before (control), and for each concentration of drug.

### Cell culture

Primary patient derived fibroblasts were isolated from prostate tissue using an established methodology described by Lawrence et al.^23^. Briefly, tissue was collected using the above methodology, placed in transfer media, and weighed. Tissue was washed in PBS supplemented with gentamicin (100 µg/mL), and cut into ~ 1 mm^2^ pieces using sterile scalpel blades. The dissected tissue was placed into RPMI1640 media containing P–S, 5% FCS, gentamicin, 225 U/mL collagenase and 125 U mL hyaluronidase and incubated in an oven (37 °C) with a rotator for 16 h (overnight). Following incubation, the tissue was processed into a single cell suspension by mechanical digestion with pipette tips, and centrifuged. The remaining cell pellet was resuspended in RPMI1640 media containing 5% FCS and P–S antibiotics and placed in a T25 flask. Following initial passage, cells were cultured in RPMI/5% FCS/1 nM testosterone/P–S and utilised at passages 4–8. WPMY-1 immortalised prostate fibroblasts were obtained as a gift from Professor Judith Clements and were utilised at passages 49–52.

#### Reagent preparation

Powdered oxytocin (Sigma Aldrich, St Louis, MO, USA) and atosiban (Sigma Aldrich, St Louis, MO, USA) were dissolved in distilled water to form a 1 mM stock concentration. Serial dilutions were performed in culture media (RMPI 1640 without Phenol Red, with 5% charcoal stripped FCS and P–S antibiotics) to ensure the final volume of reagent dissolved in distilled water added to each well was the equivalent of 0.01% total media volume. Distilled water was added at the equivalent volume of 0.01% media volume to control columns to act as a vehicle control.

#### Proliferation

Fibroblast proliferation in response to oxytocin [0.1 nM–1 µM] was determined using Cell Titre 96 Aqueous One assays (Promega, Australia). Cells were seeded into a 96 well plate at a cell density of 5 × 10^3^, and media changed to RMPI 1640 without Phenol Red, with 5% charcoal stripped FCS and P–S antibiotics to remove any proliferative effects mediated by steroid hormones. Media was changed every two (2) days for the course of the experiment. Proliferation was measured at 72 h and 120 h following initial treatment of the cells with oxytocin, with additional oxytocin of the same concentration and volume of reagent added to the media every 24 h. Atosiban (0.1 nM–1 μM) was added to media, with proliferation assessed after 72 h. Cells were incubated for one (1) hour with assay reagents, following which absorbance was measured at 490 nM, using a FLUOstar Optima plate reader (BMG, Germany). Three (n = 3) independent experiments were performed per cell line, with each experiment containing three (3) technical replicates.

#### RNA analysis

RNA was collected from cells treated with the vehicle control (0.01% distilled water), 1 nM, 10 nM and 100 nM of Oxytocin for 72 and 120 h, with additional oxytocin of the same concentration and volume of reagent added to the media every 24 h of culture. RNA was extracted using TRIzol (Invitrogen), DNase-treated and cDNA generated using Superscript III (Roche, Switzerland).

#### Qualitative RT-PCR

Qualitative real time PCR (qRT-PCR) was performed using cDNA generated as described above. Primers utilised in this experiment are listed in Table 2. PCR analysis was performed using QuantiTect SYBR Green Kit (Qiagen). Each gene was normalised to the housekeeping gene (*GAPDH*) and compared with vehicle controls using the 2^*−ΔΔC*t^ method, and fold change was calculated relative to vehicle control of independent cell lines.

**Table 2:** PCR primer sequences.

Gene	Primer sequence	Product length (base pairs)
*SMTN*	(fwd) 5′-GAGCAGACCCGAGTGAACAA-3′ (rev) 5′-CGTGCTCTGATCCAGCATCT-3′	111
*CNN1*	(fwd) 5′-TCCAGCCCCTGTAGAACTCA-3′ (rvs) 5′-GAATAGCGTTGCTCAGTGCG-3′	168
*ACTA2*	(fwd) 5′-TGTAAGGCCGGCTTTGCT-3′ (rvs) 5′-CGTAGCTGTCTTTTTGTCCCATT-3′	112
*COL1A1*	(fwd) 5′-GGGTAAGTCCCTTTCTGCCC-3′ (rvs) 5′-TTGGGTGTTTGAGCATTGCC-3′	177
*FN1*	(fwd) 5′-ACAAGCATGTCTCTCTGCCAA-3′ (rvs) 5′-GCAATGTGCAGCCCTCATTT-3′	192
*OXTR*	(fwd) 5′-GACTCGGTGCAGTGGAAGC-3′ (rvs) 5′-CTCCTCTGAGCCACTGCAAA-3′	108
*GAPDH*	(fwd) 5′GCAAATTCCATGGCACCGT-3′ (rvs) 5′-TCGCCCCACTTGATTTTGG-3′	106

### Statistical analysis

The half-maximal (EC_50_) value was calculated using GraphPad Prism version 7.05 for Windows (GraphPad Software, La Jolla California USA). Unless noted, all data are mean ± SEM, with statistical analysis performed by Student's *t*-test, one-way ANOVA and Dunnets post hoc test, or two-way ANOVA and Tukeys post hoc test, using GraphPad Prism version 7.05 software with significance deemed at *p* < 0.05. A generalized Extreme Studentized Deviate (ESD) test with an Alpha level of 0.05 was performed to exclude outliers. Multi-linear regression analysis was used to correlate clinical records with the percentage response to pharmacotherapy treatment using IBM SPSS Statistics for Windows, Version 22.0 (IBM Corp: Armonk, NY).

## Results

### Oxytocin receptor is widely expressed in the human prostate, and is co-localised with contractile cells in the prostate stroma

**Figure 1:**
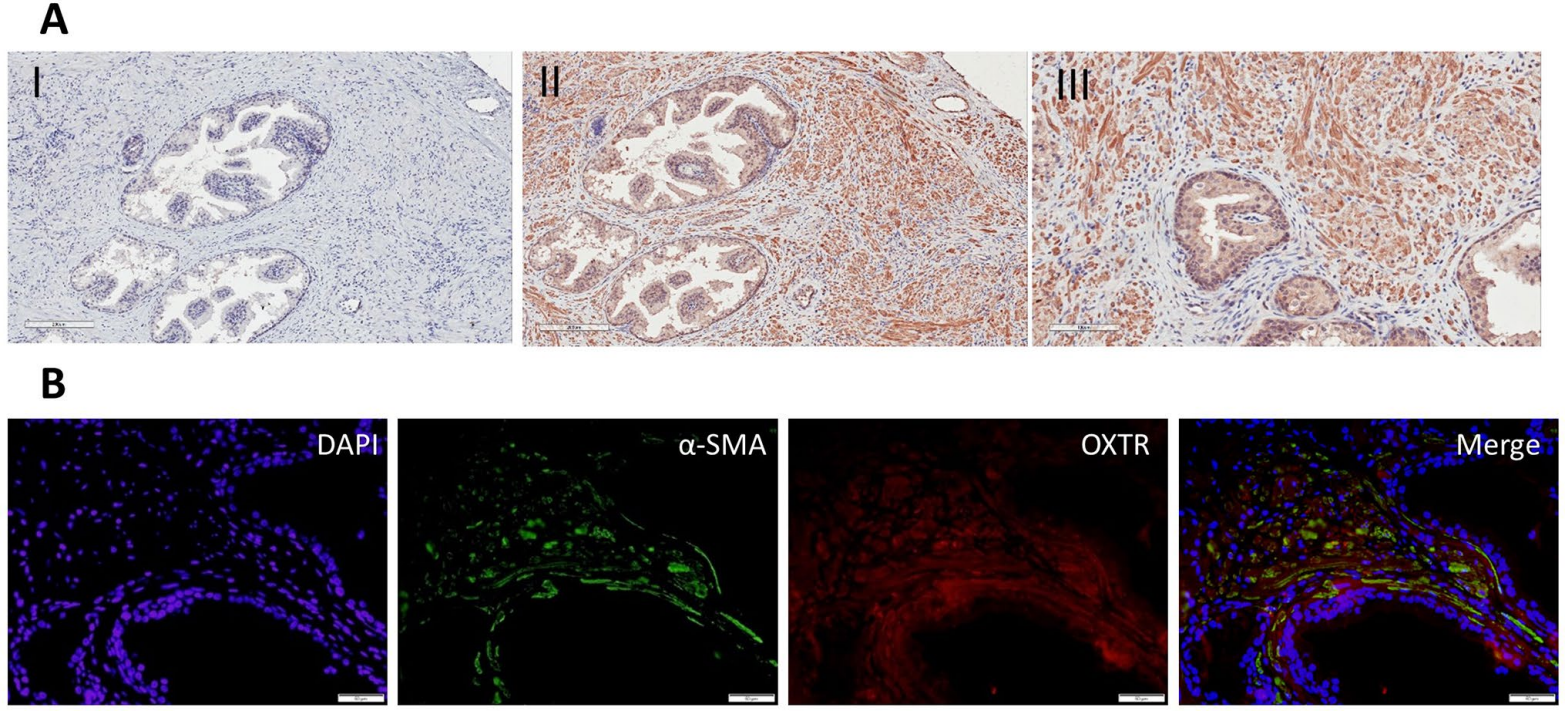
The oxytocin receptor is widely expressed in the human prostate and is co-localised with contractile stromal cells. Representative images of sections of human prostatic transition zone specimens with (**A**) immunohistochemistry staining of (I) negative control (scale bar = 200 μm), and (II, III) oxytocin receptor (OXTR) (scale bar = 200 μm and 100 μm respectively), and (**B**) Immunofluorescence staining of DAPI, α-Smooth Muscle Actin (α-SMA), and OXTR. Merged image demonstrates co-localisation (yellow) (scale bar = 50 μm).

Immunohistochemistry was conducted to assess the presence and distribution of OXTR. Specificity of staining was determined by substituting the primary OXTR antibody for goat immunoglobulin, diluted to 1.25 μg/mL (Fig. 1A, I). OXTR expression was observed in both epithelial and stromal cells (Fig. 1A, II & II). The epithelial compartment demonstrated consistent staining for the OXTR in all visible glands (observed by brown DAB staining), with no noticeable difference observed visually in the staining of basal or luminal cells. To determine if OXTR was expressed on contractile cells, double immunofluorescence staining was performed for OXTR and α-Smooth Muscle Actin (α-SMA), a well-established cytoskeleton protein of contractile cells. Co-localization between OXTR and α-SMA was observed (Fig. 1B).

### Oxytocin did not modulate the proliferation of prostatic stromal cells, but upregulated the mRNA expression of OXTR and genes related to smooth muscle differentiation

**Figure 2:**
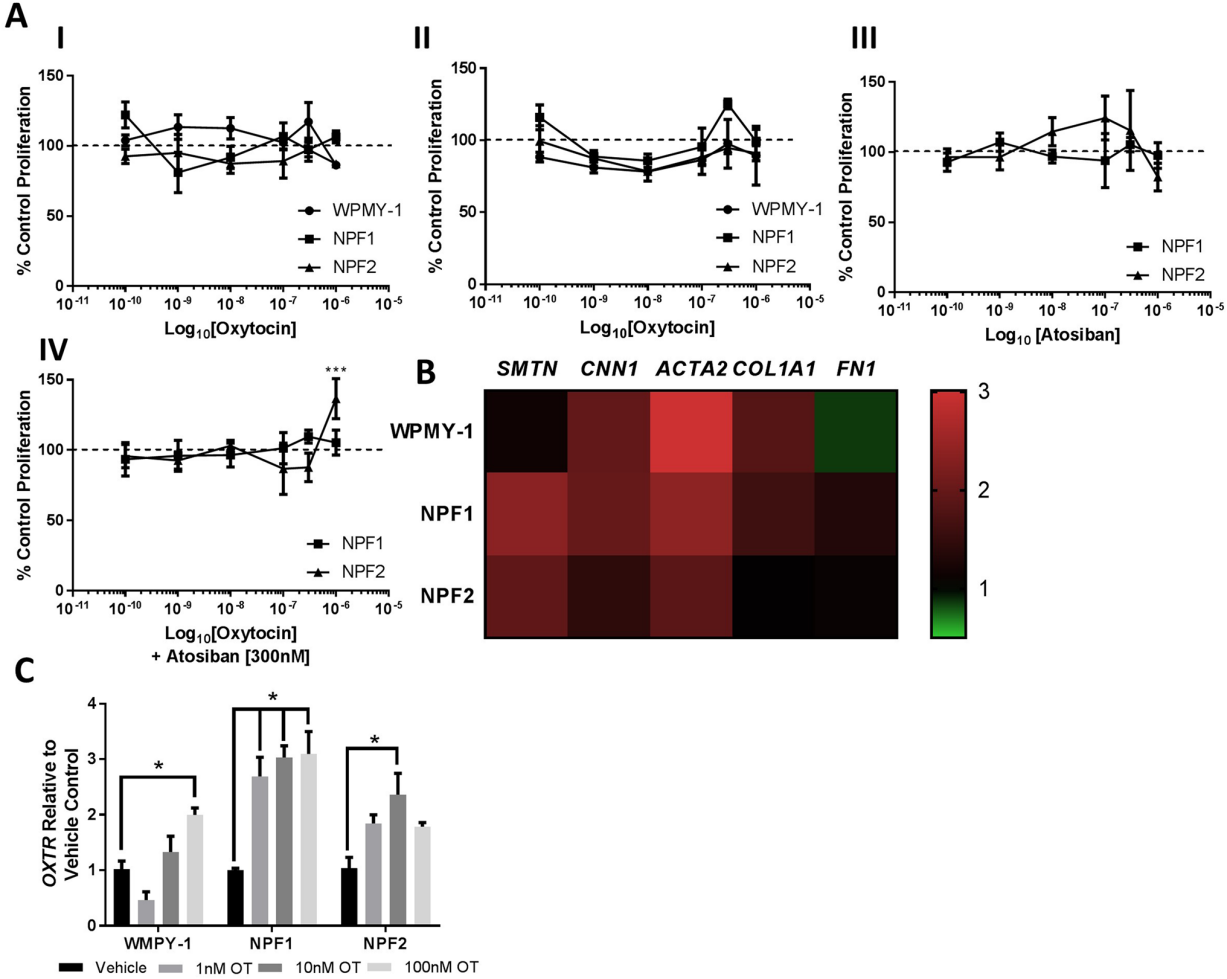
Exogenous oxytocin did not induce proliferation of stromal cells in culture. (**A**) To assess the static component, isolated primary patient derived non-malignant (NPF1 and NPF2) and immortalised (WMPY1) human prostate fibroblasts were exposed to (I) exogenous oxytocin (0.1 nM–1 μM) for 72 h, exogenous oxytocin (0.1 nM–1 μM) for 120 h (III) the OXTR antagonist Atosiban (0.1 nM–1 μM), or (IV) increasing concentrations of exogenous oxytocin (0.1 nM–1 μM), in the presence of Atosiban (300 nM) in culture for 72 h, following which proliferation was assessed using Cell Titre 96 Aqueous One cellular proliferation assay, with proliferation normalised to the vehicle control of each cell line (n = 3), unpaired one-way ANOVA with Dunnet’s multiple comparisons test, ***p ≤ 0.001). (**B**) RNA was harvested from cells treated with Oxytocin (10 nM) for 72 h and qualitative RT-PCR was used to determine the fold change in mRNA transcripts from vehicle control for genes involved in myofibroblast differentiation. (III) fold change in Oxytocin Receptor (OXTR) mRNA transcript levels normalised to vehicle control (n = 3, unpaired one-way ANOVA with Dunnet’s multiple comparisons test, *p ≤ 0.05).

To assess the effect of exogenous oxytocin on the static component of BPH, an immortalised and two primary derived prostate fibroblasts were exposed to oxytocin at increasing concentrations [0.1 nM–1 μM]. Proliferation was assessed using a commercially available proliferation assay 72 or 120 h following initial exposure to oxytocin, and proliferation normalised to the vehicle control (% control proliferation). There was no significant difference in proliferation at any concentration of oxytocin (Fig. 2A, I & II). Primary patient derived fibroblasts (NPF1 & NPF2) were additionally exposed to atosiban [0.1 nM–1 μM], an OXTR antagonist for 72 h, with no significant changes in proliferation observed (Fig. 2A, III). Proliferation was significantly increased from vehicle control in NPF2 cells treated with oxytocin [1 μM] and atosiban [300 nM] simultaneously, although this result was not replicated with NPF1 cells, or at lower concentrations of oxytocin (Fig. 2A, IV).

RNA was harvested from cells at the same time point, and qualitative RT-PCR was used to assess the fold change of the genes *SMTN, CNNI1, ACTA2, COL1A* and *FN1* in cells exposed to oxytocin [10 nM] for 72 h. *SMTN* and *CNN1* encode the genes smoothelin and calponin, markers of prostate smooth muscle differentiation, and both were upregulated ~ twofold in primary patient derived fibroblasts, but not in the immortalised line (Fig. 2B). *ACTA2* encodes α-SMA, which is a marker of both myofibroblast and smooth muscle differentiation and was ~ 2–2.5 fold upregulated in all cell lines. The fibroblast markers *COL1A,* which encodes Collagen I, was elevated by 1.8-fold in the WPMY-1 cell line, but not in the two patient derived fibroblasts. *FN1,* which encodes Fibronectin, was not upregulated in any cell line following exposure to oxytocin (Fig. 2B). Exposure to oxytocin [10 nM] induced a ~ two- to three-fold increase in mRNA transcripts of the *OXTR* gene across all three cell lines (Fig. 2C).

### Oxytocin induced an increase in the frequency of spontaneous contractions of the human prostate

**Figure 3:**
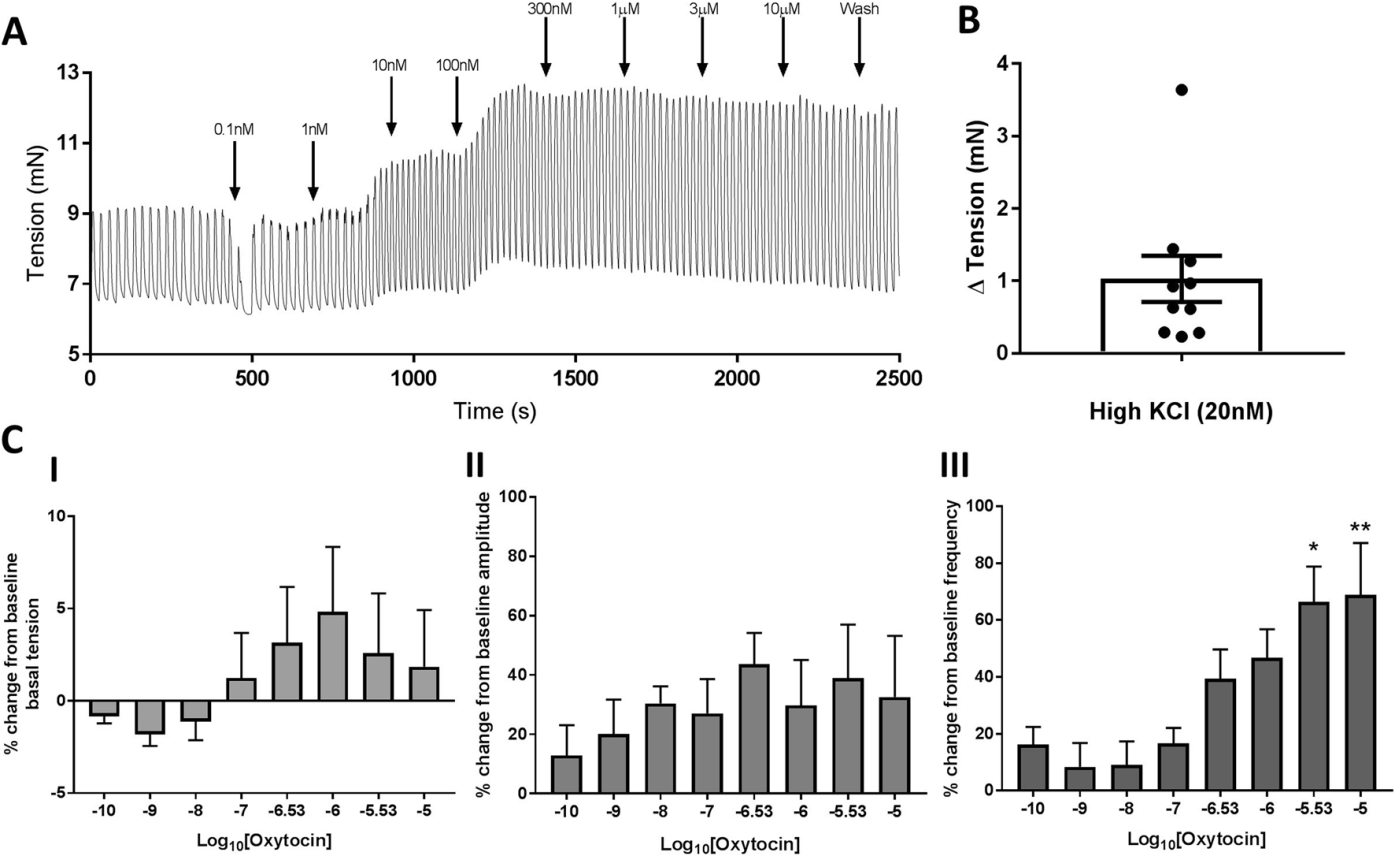
Application of exogenous oxytocin modulated the dynamic component by significantly increasing the frequency of spontaneous contractions within the human prostate. To assess the dynamic component, primary human prostate tissue collected from the transition zone (TZ) was exposed to increasing concentrations of exogenous oxytocin (0.1 nM–10 μM). (**A**) representative trace of spontaneous contractions following exposure to exogenous oxytocin (0.1 nM–10 μM). (**B**) Application of High KCl (20 mM) induced a robust contraction following conclusion of the dose response curve, confirming tissue viability. The percentage change (% change) from baseline (I) basal tension, (II) amplitude and (III) frequency of spontaneous contractions following application of exogenous oxytocin (0.1 nM–10 μM) (n = 9, paired one-way ANOVA with Dunnets multiple comparisons test, *p < 0.05, **p < 0.01).

To assess the effect of exogenous oxytocin on the dynamic component of BPH, primary human prostate tissue was collected from the Transition Zone (TZ) of men undergoing radical prostatectomy, and contractility was assessed in vitro using organ bath techniques. Specimens were exposed to cumulative concentrations of exogenous oxytocin [0.1 nM–10 μM] (Fig. 3A, I), with the average basal tension (mN), amplitude (N/g) and frequency (min^−1^) of spontaneous contractions assessed, and converted to a percentage of control spontaneous activity (Fig. 3B, I, II & III respectively). Tissue viability was assessed following a washout period after the completion of the dose–response curve by exposing tissue to potassium chloride (KCl; 20 mM), which induced a robust contraction in all preparations (Fig. 2B). Oxytocin did not significantly modulate basal tension or amplitude, but did significantly (*p* < 0.05) upregulate the frequency of spontaneous contractions.

### Interpatient variability in the increase in frequency of spontaneous contractions was correlated with age

**Figure 4:**
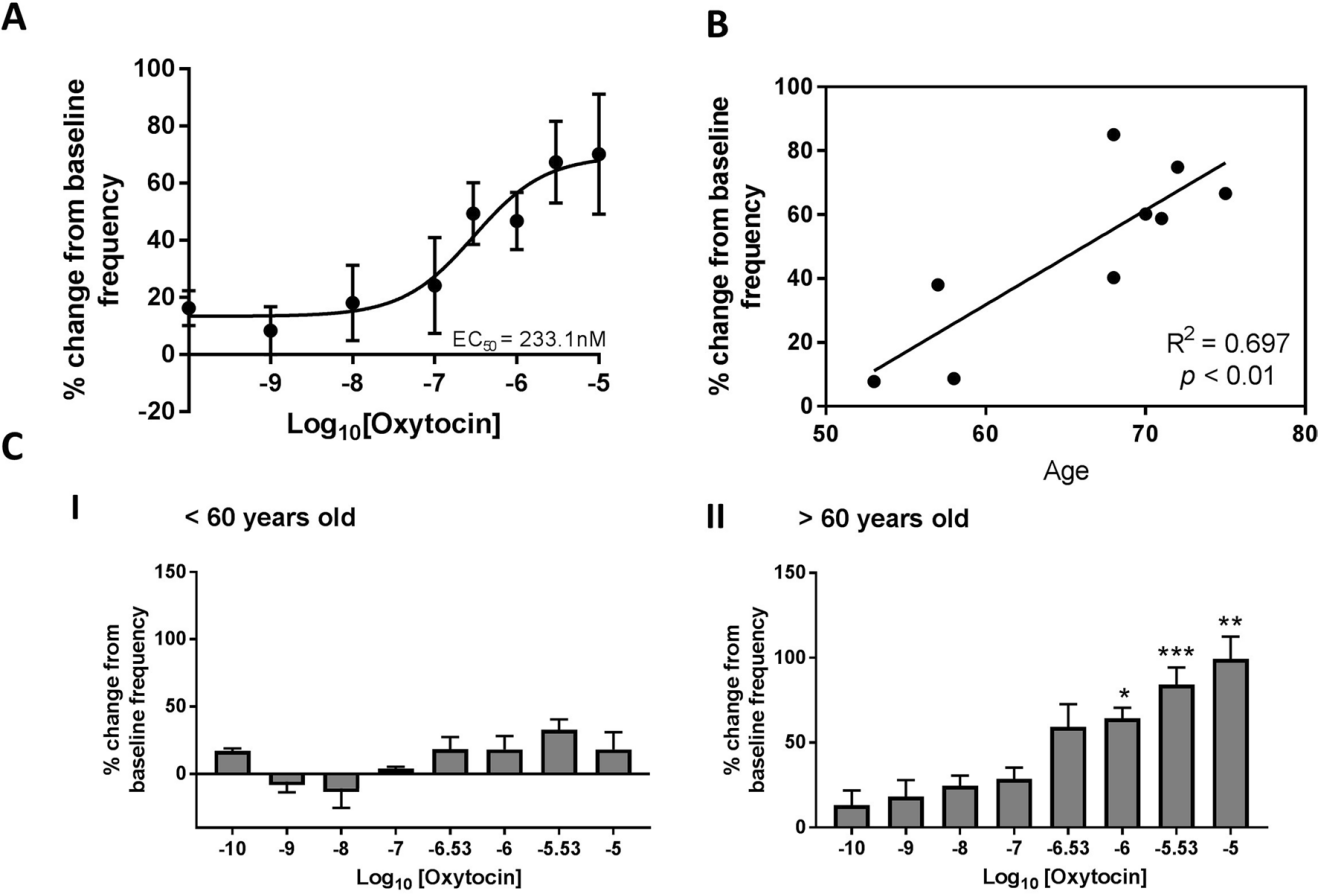
Variability in oxytocin induced change in spontaneous contraction frequency is significantly correlated with age. (**A**) Dose response curve of % change from baseline frequency with increasing concentration of exogenous oxytocin (n = 9, non-linear regression). (**B**) Correlation between % change from baseline frequency and age (n = 9, Linear Regression analysis, *p* < 0.05 considered significant). (**C**) % change from baseline frequency in response to increasing concentrations of exogenous oxytocin from prostate tissue collected from men (I) less than 60 years old (n = 3), and (II) greater than 60 years old (n = 6) (paired one-way ANOVA with Dunnets multiple comparisons test, *p < 0.05, **p < 0.01, ***p < 0.001).

The half maximal (EC_50_) of exogenous oxytocin was determined by non-linear regression to occur at 233.1 nM (Fig. 4A). However, there was a notable interpatient variability in the percentage change from baseline frequency. Linear regression analysis was performed against percentage change from baseline frequency at 1 μM, and patient clinical parameters. A significant association was observed between percentage change and patient age at time of radical prostatectomy (Fig. 4B). Division of the cohort into groups under (n = 3) and over (n = 6) 60 years of age indicated that older patient tissue had a significant (p < 0.05) increase in the frequency of spontaneous contractions (Fig. 4C, I), and this was not observed in tissue from men under 60 years old (Fig. 4C, II).

### Atosiban significantly attenuated spontaneous contractile parameters

**Figure 5:**
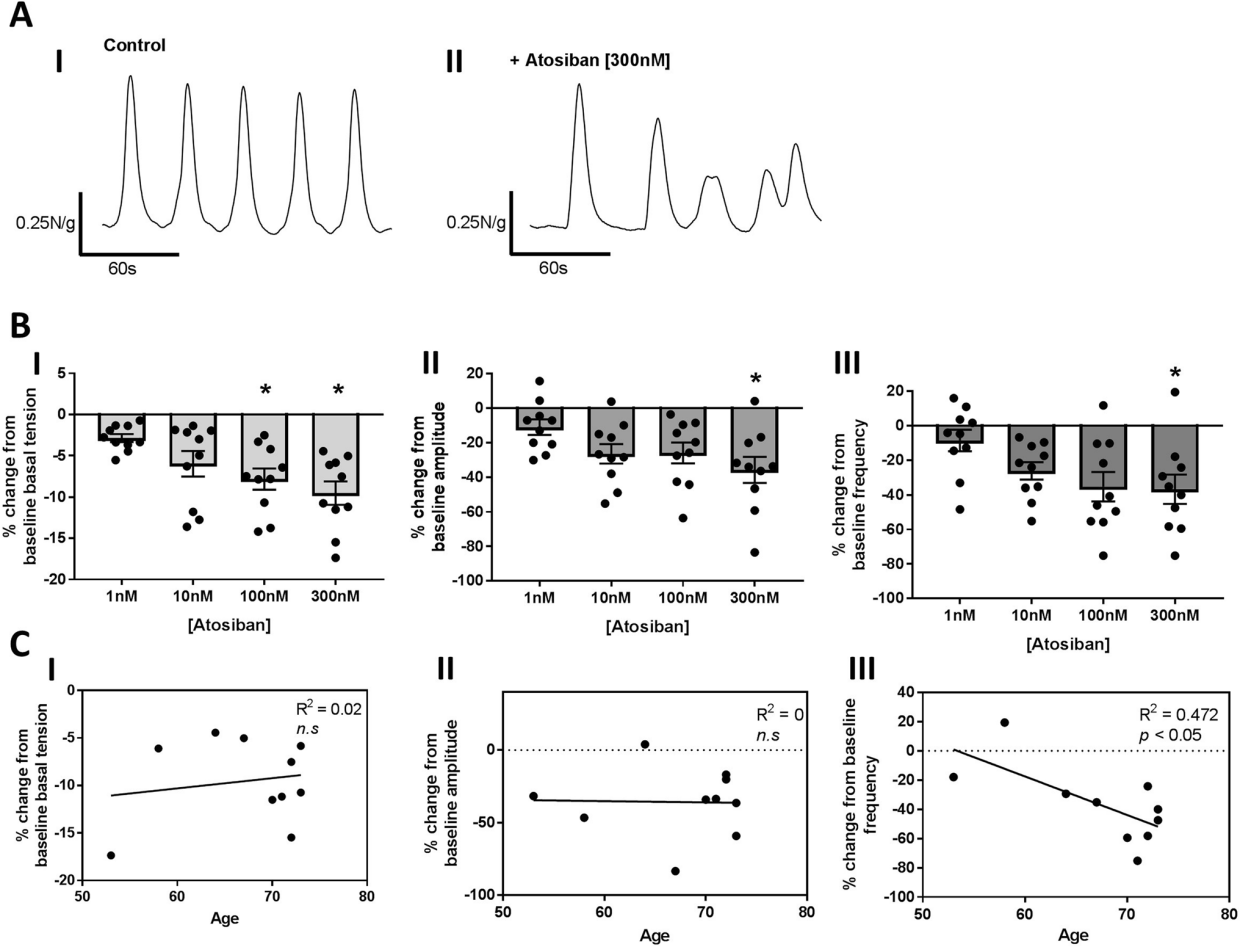
Application of atosiban, an OXTR antagonist, significantly reduced spontaneous contractile parameters in human prostate specimens, with reduction in frequency correlated with age. (**A**) Representative trace of spontaneous contractions (I) prior to and (II) following application of atosiban [300 nM]. (**B**) Average % change from baseline (I) basal tension, (II) amplitude, and (III) frequency of spontaneous contractions following incubation with increasing concentrations of atosiban [1, 10, 100 and 300 nM] (n = 10. paired one-way ANOVA with Dunnets multiple comparisons test, *p < 0.05, **p < 0.01). (**C**) correlation between average % change from baseline (I) basal tension, (II) amplitude, and (III) frequency of spontaneous contractions following incubation with atosiban [300 nM] with age (n = 10, Linear Regression analysis, p < 0.05 considered significant).

To determine its potential as a BPH pharmacotherapy, unstimulated sections of human prostate were incubated with increasing concentrations of atosiban, an OXTR antagonist [1, 10, 100 and 300 nM]. Sections had not been exposed to any exogenous stimulus prior to incubation with atosiban. The basal tension of spontaneous contractions was significantly (*p* < 0.05) reduced at 100 nM and 300 nM (Fig. 5B, I), with amplitude and frequency (Fig. 5B, II & III respectively) also significantly attenuated at 300 nM of atosiban (*p* < 0.05).

### Interpatient variability in the decrease in the frequency of spontaneous contractions was correlated with age

Regression analysis was performed against the percentage decrease of all three parameters at atosiban [300 nM] and patient age. A significant (R^2^ = 0.472, p < 0.05) association was observed between percentage change from baseline frequency and age, indicating tissue from older patients had a greater reduction in the frequency of spontaneous contractions (Fig. 5C, III). No association was observed between age and percentage change from baseline basal tension or amplitude (Fig. 5C, I & II respectively).

## Discussion

Our study investigated the role of the peptide hormone oxytocin in prostate physiology, and is the first study to comprehensively describe the effects of oxytocin and Atosiban on spontaneous contractility of the human prostate. While no significant effects of oxytocin on proliferation of stromal cells was observed, treatment of primary prostatic fibroblasts expressing the OXTR upregulated mRNA transcripts of genes associated with smooth muscle differentiation. These results support the intriguing possibility of OXTR antagonists as a pharmaceutical agent for the treatment of BPH.

We have previously demonstrated that the frequency of myogenic contractions are significantly upregulated in men with clinically diagnosed BPH^17^, and hypothesised that changes to inherent myogenic contractility are driven by intrinsic factors within the prostate, notably oxytocin levels. The presence of the OXTR in the human prostate was previously described^9^ and confirmed in this study (Fig. 1). Dual staining immunofluorescence revealed co-localization of OXTR with α-SMA (a marker of contractile filaments), supporting our hypothesis that oxytocin is a modulator of contractility within the prostate. Herbert et al., previously reported weak OXTR staining in younger men (9 and 18 years old), which increased with age (28 and 33 years old) and in BPH patients. However, they did not comment about which compartment (epithelial or stromal) the intensity of staining was increased in, rather they reported an overall increase^11^. A future direction would be to identify specifically which compartment of the prostate OXTR expression increases in with age, as we were unable to address this due to our cohort of older patients (> 53 years old).

The application of exogenous oxytocin significantly increased the frequency of spontaneous contractions in a dose-dependent manner. Assinder and Nicholson determined that explants of human prostate produced endogenous oxytocin^24^, suggesting that oxytocin is a paracrine regulator of spontaneous smooth muscle tone in the human prostate. Significantly, we also demonstrate that application of atosiban, an OXTR antagonist, reduced the basal tension, amplitude, and frequency of spontaneous contractions by 9.52 ± 1.42%, 34.7 ± 6.9% and 34.9 ± 7.8% respectively at a concentration of 300 nM. Atosiban was applied to unstimulated sections of human prostate, supporting the hypothesis that oxytocin endogenously contributes to prostatic smooth muscle tone.

Correlation analysis between changes in the frequency of spontaneous contractions in response to both oxytocin and atosiban indicate that tissue collected from older men had greater responsiveness compared to younger men within the cohort. As the percentage of men suffering from BPH significantly increases with age^2^, a pharmaceutical agent that is more effective in older men is of clinical interest. We postulate the age-related differences found within our studies are likely a reflection of changes in the expression of the OXTR within the transition zone of the human prostate. Our results are consistent with an earlier publication by Herbert et al., which noted an increased distribution of OXTR in human prostate tissue collected from older men, compared to younger controls^11^. We hypothesise this mechanism is likely to occur as a result of the changing hormonal balance within the prostate as men age, with testosterone levels decreasing while estrogen levels remain steady^25^. In the uterus, increasing estrogen concentrations are associated with an upregulation in OXTR expression^26^. However, further studies are needed to determine if estrogen induces OXTR upregulation in the human prostate. A future avenue to investigate this would be to examine the expression of OXTR and responsiveness of prostate tissue to oxytocin from men treated with 5α-reductase inhibitors, as this treatment alters the hormone balance within the prostate, and may interact and alter oxytocin signalling. Another possible mechanism is that increased oxytocin may regulate changes in expression of the OXTR. In isolated stromal cells, exposure to 10 nM of exogenous oxytocin increased mRNA expression of the OXTR by ~ two- to three-fold after 72 h in culture. Therefore, increasing levels of oxytocin may positively regulate the expression of the OXTR, leading to increased sensitivity in older men.

Whittington et al. reported that oxytocin reduced the proliferation of primary prostate fibroblasts^16^, in contrast to Xu et al., who reported that oxytocin induced proliferation of the immortalised cell lines RWPE-1 (prostate epithelium) and WPMY-1 (prostate fibroblasts) through the MAPK/ERK pathway^10^. A recent publication by Li et al. reported increased proliferation in WPMY-1 cells in response to oxytocin, with significant results observed after 5 days treatment with 10 nM of oxytocin^27^. Our study did not find a significant impact of oxytocin on fibroblast proliferation. We hypothesise this is likely due to different methodology used between these studies, as we replenished media with additional oxytocin every 24 h to account for the short biological half-life of the hormone and used both primary and immortalised cell lines to compare differences in response. As WPMY-1 cells are an immortalized line, we used these cells at a much higher passage (49–52) than our primary cell lines (maximum passage number of 8). There is a higher risk of the WPMY-1 cell line undergoing genotypic and phenotypic variation over an extended period, and as such may not adequately represent primary cells^28^. It is unclear at which passage Li et al. obtained the WPMY-1 cell line, hence offering a possible reason for the differing results. However, across all three (3) lines, we did not observe any significant differences in the rate of proliferation after 72-h of culture.

Fibroblasts and stromal cells play an important role in the initiation and progression of BPH^29^. Schauer and Rowley propose that BPH arises because of a “reactive stroma”, predominantly caused by myofibroblasts^30^. Primary stromal cultures isolated from the human prostate contain a mixture of fibroblast, myofibroblasts, and smooth muscle cells, which have been demonstrated to differentiate to another phenotype in cell culture^31^. In the fibroblast lines utilised in this study, all cells express vimentin but show a heterogenous expression of α-SMA, indicating a mixed line of fibroblast and myofibroblasts. By treating fibroblasts with 10 nM of exogenous oxytocin, we observed an upregulation of markers of myofibroblast differentiation, notably *ACTA2,* which was ~ 2–2.5 fold upregulated at the mRNA level. While the trend between cell lines was different, overall, the data suggests that oxytocin induces a change in the genes involved in the differentiation state of stromal cells. The higher endogenous levels of oxytocin in the prostate observed by Assinder and Nicholson may promote the differentiation from a fibroblast or myofibroblast to a smooth muscle phenotype, augmenting disease progression by promoting a reactive stroma. Therefore, lowering endogenous oxytocin may be an important pharmacological target for reducing the quantity of reactive stroma and smooth muscle/tone cells within the prostate.

OXTR antagonists are of interest in the treatment of premature ejaculation (PE), and show promising preliminary results in animal models^32^. Cligosiban (IX-10) was recently determined by a Phase II, randomised, double-blind, placebo-controlled study to be effective in treating PE^33^. As PE is a common co-morbidity with BPH^34^, OXTR antagonists may be of dual benefit in the treatment of both diseases.

A limitation of our study is that we did not investigate the role of epithelial or co-cultured cells in our cell culture experiments. However, the focus of our investigation was on the role of oxytocin in the prostate stroma, as it is the compartment that most contributes to the pathogenesis of BPH^35^. Our cell culture experiments were designed to address the contentious findings between previous publications^10,16,27^ with regards to the proliferative effects of oxytocin on prostate fibroblasts by using a robust methodology and both primary and immortalised cell lines. Hence, the focus of our paper was not on the epithelium, although epithelial-stromal crosstalk is an interesting avenue to consider further due to the presence of OXTR in both compartments of the prostate.

Both an advantage and a limitation of our study was the use of prostate cancer patients to investigate non-malignant prostate disease. By selecting for low-grade patients with small tumours, we were able to obtain high quality prostate specimens directly from the TZ. Previous attempts to obtain tissue directly from BPH patients undergoing Transurethral Resection of the Prostate (TURP) resulted in poor quality, often burnt, samples, which were unsuitable for organ bath experiments. The average prostate volume (39.46 cc ± 9.09) in our cohort is greater than the definition of an enlarged prostate (30 cc)^36^, indicating that most of the cohort had an enlarged prostate at time of prostatectomy. However, as these men were treated for prostate cancer, we have limited information regarding their overall BPH symptomology, such as IPSS scores, urine flow rate, and BMI, which limited the correlation analysis we were able to perform to age and prostate volume. As age, prostate volume and BPH progression are so intrinsically linked^2^, there is a causality dilemma in our findings; while the majority of our findings are an assessment of prostate tissue function in the presence of oxytocin and an OXTR, the age and prostate enlargement in our cohort means the relevance of our findings to the BPH disease state are significant.

Overall, BPH is a highly prevalent disease of the ageing male population, with limited pharmaceutical interventions available. Our report highlights the importance of oxytocin as a paracrine regulator of the dynamic component of BPH, particularly in upregulating the frequency of spontaneous contractions in the human prostate. This study provides a rationale for further investigation into the use of OXTR antagonists for the clinical treatment of BPH.
